# Human Fecal Transplantation Modifies the Gut Microbiota but Not Metabolites in Colon Cancer Patient-Derived Xenografts

**DOI:** 10.3390/ijms27031438

**Published:** 2026-01-31

**Authors:** Katarzyna Unrug-Bielawska, Zuzanna Sandowska-Markiewicz, Ewelina Kaniuga, Magdalena Cybulska-Lubak, Monika Borowa-Chmielak, Paweł Czarnowski, Magdalena Piątkowska, Aneta Bałabas, Krzysztof Goryca, Natalia Zeber-Lubecka, Maria Kulecka, Michalina Dąbrowska, Piotr Surynt, Małgorzata Statkiewicz, Izabela Rumieńczyk, Michał Mikula, Jerzy Ostrowski

**Affiliations:** 1 Department of Genetics, Maria Sklodowska-Curie National Research Institute of Oncology, Roentgena 5, 02-781 Warsaw, Poland; 2 Department of Gastroenterology, Hepatology and Clinical Oncology, Centre of Postgraduate Medical Education, Roentgena 5, 02-781 Warsaw, Poland

**Keywords:** fecal microbiota transplantation (FMT), FOLFOX treatment, colorectal cancer patient-derived xenograft (CRC PDX), stool microbiota, stool metabolites

## Abstract

Gut microbiota influences colorectal cancer (CRC) development, tumor progression, and response to therapy. Fecal microbiota transplantation (FMT) has been proposed as a strategy to restore microbial balance and modulate treatment outcomes. We evaluated the effects of human fecal transplantation on gut microbiota composition, metabolites, tumor growth, and the efficacy of folinic acid, fluorouracil and oxaliplatin (FOLFOX) chemotherapy in four CRC patient-derived xenograft (CRC PDX) models in NSG mice. Gut microbiota was profiled by 16S rRNA sequencing; short-chain fatty acids (SCFAs) and amino acids (AAs) were analyzed by mass spectrometry. Prolonged FMT significantly altered gut microbiota structure, increasing α-diversity and modifying β-diversity, and induced distinct changes in bacterial genera. FMT alone did not affect tumor growth. FOLFOX inhibited tumor progression in all CRC PDXs, with FMT enhancing therapeutic efficacy in two models. Despite substantial microbiota shifts, FMT exerted minimal or no effect on fecal SCFAs and AAs. FMT induced robust microbiota remodeling but did not modify selected stool metabolites or intrinsic tumor growth. However, FMT enhanced FOLFOX responsiveness in selected CRC PDXs, supporting a microbiota-mediated modulation of chemotherapy outcomes.

## 1. Introduction

Colorectal cancer (CRC) is the third most common cancer and the fourth leading cause of cancer-related mortality [1]. The development of CRC relates to changes in lifestyle, diet, inflammation and microbiome [2]. The gut microbiota extracts nutrients and energy from the diet, trains the immune system, protects itself from opportunistic pathogens and produces local and systemic metabolites. Microbial community complex imbalances (dysbiosis) affect the regulation of epithelial cell proliferation by changing endogenous metabolites and microbial products such as short-chain fatty acids (SCFAs), amino acids (AAs), secondary bile acids, and lipopolysaccharides [3]. Although it is not possible to define a healthy baseline microbiome, a dynamic balance involving bacterial diversity and functional redundancy without the predominance of a single strain is critical for maintaining the normal function of the intestine. In the case of intestinal dysbiosis, changes in the commensal balance can lead to pathobiomes [4,5].

The intestinal microorganisms are closely associated with the development, progression and response of various cancers to treatment. This process is mediated through numerous causes, including the transformation of the host genome, alterations in DNA stability caused by viruses, metabolic abnormalities, and inappropriate activation of the immune system [6]. Although tumors change the composition of intestine microbes, a combination of microbes and nutritional compounds can prevent the development of bowel polyps in animals and humans with this [7,8,9,10]. In many human and animal studies, the beneficial or harmful effects of specific bacterial strains and metabolites associated with intestinal microorganisms, such as biliary acids and SCFAs, are reported [8,11]. Oncological treatments such as surgery, chemotherapy, immune therapy, and radiotherapy can lead to bacterial dysbiosis which may reduce the effectiveness and increased toxicity of therapy [12]. Thus, the restoration of normal microflora is becoming a promising research guide for the prevention and treatment of cancer. Since symbiotic intestinal microorganisms can improve the effectiveness and reduce the toxicity of chemotherapy, immunotherapy, and radiotherapy, probiotics and the transplantation of normal feces are effective methods for restoring “normal” intestinal microbiota in patients with gastrointestinal cancer [13,14]. Fecal microbiome transplantation (FMT) from a healthy donor into the recipient’s intestinal tract to restore the dysbiotic microbiota is mostly used to treat *Clostridioides difficile* infections and is also being tested in the treatment of other diseases including cancer [15].

FOLFOX is a combination of 5-fluorouracil, oxaliplatin, and folic acid, and is a standard treatment for advanced CRC on the first line [16]. However, FOLFOX has altered the composition of gastrointestinal microorganisms in mice with CRC xenograft tumors [8] and in mice implanted with syngeneic CT26 adenocarcinoma cells [17]. In these models, FMT reduced the severity of diarrhea and intestinal mucositis, indicating the restoration of the gut microbiota composition [17]. However, the evidence supporting FMT’s effectiveness in improving chemotherapy response remains unclear. In the present study, we investigated the effect of fecal transplantation from healthy humans into immunodeficient NOD scid gamma (NSG) mice grafted with human colon adenocarcinoma patient-derived xenografts (CRC PDX) on tumor growth and the efficacy of FOLFOX treatment by analyzing the fecal microbiota and changes in metabolite composition.

## 2. Results

### 2.1. Effects of FMT on Body Weight and Gut Microbiota at Two Months (T1)

**Figure 1:**
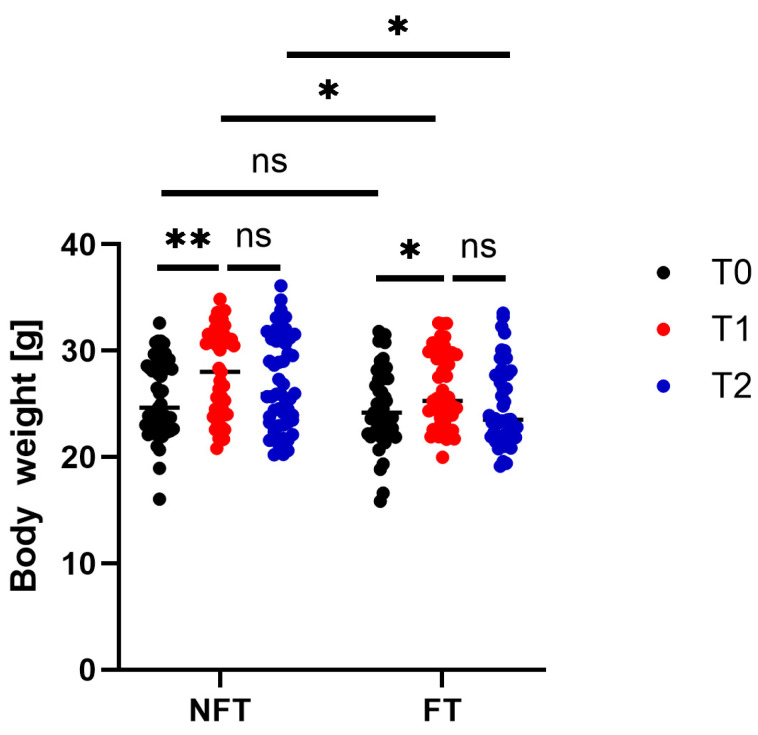
Body weights of mice that underwent fecal transplantation (FT) (*n* = 49) and those that did not (NFT) (*n* = 53) at the start time point (T0) of the experiments, after two months of fecal microbiota transplantation (T1), and at T2 (the early growth of patient-derived xenografts from human colon adenocarcinoma). Asterisks indicate significant differences; * adjusted *p* < 0.05, ** adjusted *p* < 0.01, ns—*p* > 0.05.

As shown in Figure 1, at the start time point (T0) of the experiments, the body weight of the mice was comparable between the two groups. At the T1 time point (after two months of fecal microbiota transplantation (FMT)), both groups of mice showed a significant increase in body weight compared to T0. At T2 (4–8 weeks after implantation of xenografts derived from human colon adenocarcinoma patient (CRC PDX); early tumor growth), the body weight of both no fecal transplanted (NFT) and fecal transplanted (FT) mice did not differ compared to those at T1. At T1 and T2 time points, the body weights of FT mice were significantly lower compared to NFT mice (Figure 1).

**Figure 2:**
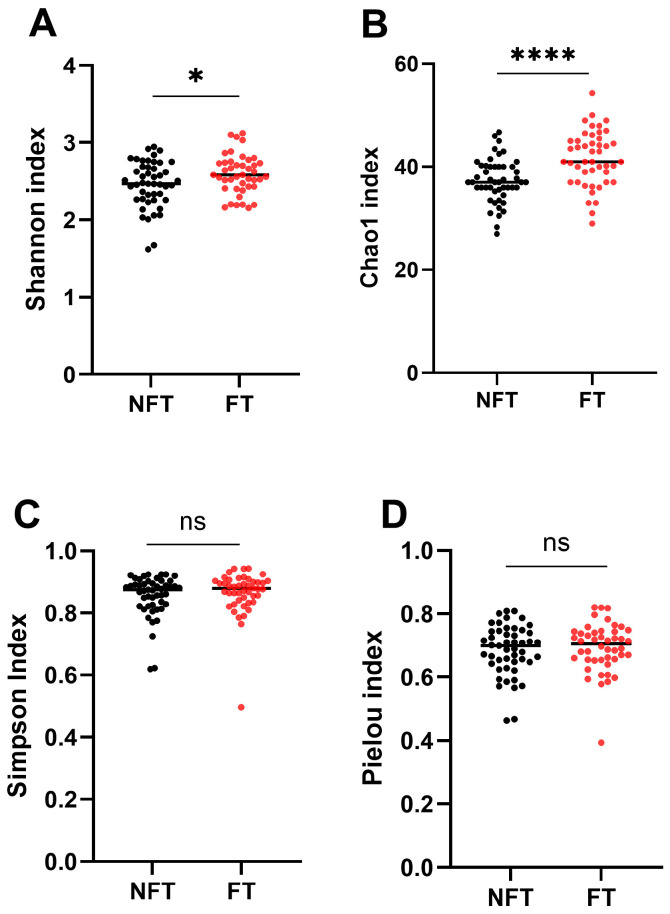
α-diversity analyzed by the Shannon (**A**), the Chao1 (**B**), the Simpson (**C**) and Pielou (**D**) indices in fecal samples collected at T1 from NFT (*n* = 51) and FT (*n* = 46) mice. The results were compiled from four separate experiments. Statistical significance: ns—adjusted *p* > 0.05; * adjusted *p* < 0.05; **** adjusted *p*  <  0.0001.

An average of 9521 reads were generated per sample (range: 1230–30,404), and analyses of the gut bacterial community structure identified several modifications. Of four α-diversity indices, the Shannon index, a marker of bacterial richness and evenness, and the Chao1 index, a marker of bacterial richness, increased significantly at T1 in FT mice compared with NFT mice (Figure 2A,B), whereas the Simpson’s diversity index that quantifies the biodiversity of a community and Pielou’s evenness index that quantifies the distribution of individuals among species in a community did not differ FT mice from NFT mice (Figure 2C,D).

**Figure 3:**
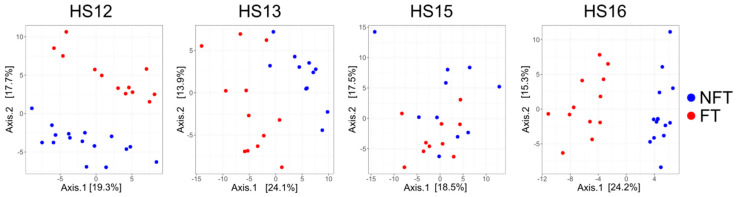
Principal coordinate analysis of fecal samples collected at T1. Each dot represents a single sample, and NFT and FT mice are shown in blue and red, respectively.

The β-diversity of the structure of the gut bacterial community, visualized by principal co-ordinate analysis (PCoA), showed a variable distribution between the groups in the experiments (Figure 3) with significant changes between the experiment batches (*p*-value = 0.0001). After two months of FMT (at T1), a clear separation between FT and NFT mice was visible in each of the experiments (Figure 3) with significant differences (PERMANOVA *p*-value: 0.006).

Taxonomic analysis at the genus level identified 28 and 24 different taxa between T0 and T1 FT and NFT groups, respectively (Tables S1 and S2). Of these, 16 differentially abundant genera were common to the two groups (A2, *Bacteroides*, *Lachnospiraceae* NK4A136 group, [*Eubacterium*] *xylanophilum* group, *Colidextribacter*, *Muribaculum*, *Alistipes*, *Turicibacter*, *Tyzzerella*, *Lachnospiraceae UCG-001*, *Clostridium* sensu stricto 1, *Lachnospiraceae UCG-008*, *Parasutterella*, *Lachnospiraceae UCG-006*, *Faecalibaculum*, and *Butyricicoccus*), whereas 12 genera were unique to the FT group (*Parabacteroides*, *ASF356*, *Anaerotruncus*, *Paraprevotella*, *Erysipelatoclostridium*, *Butyricimonas*, [*Eubacterium*] *brachy* group, *Bilophila*, *Enterorhabdus*, *Oscillibacter*, *Acetatifactor*, and *Blautia*), and eight genera were unique to the NFT group (*Bifidobacterium*, *Anaerostipes*, [*Eubacterium*] *siraeum* group, [*Eubacterium*] *oxidoreducens* group, *Pseudomonas*, *Escherichia*-*Shigella*, *Candidatus*, *Arthromitus*, and *Mucispirillum*).

**Figure 4:**
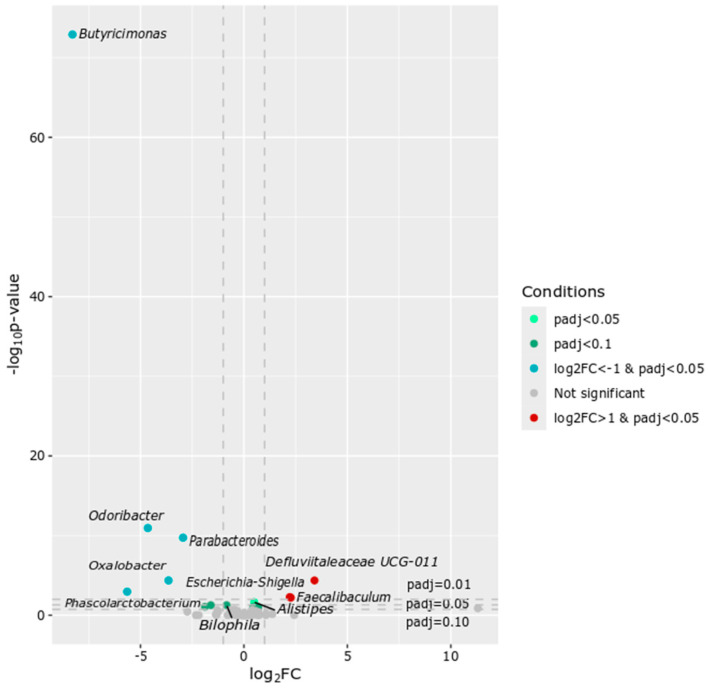
Volcano plot showing differential abundance of taxa. Each point represents a taxon, plotted by log2 fold change (log2FC) and statistical significance (−log10 *p*-value). The vertical grey dotted lines indicate the log2 fold change (log2FC) thresholds of −1 and 1.

Of 11 genera that differed between the two groups at T1, seven genera were over-represented (*Butyricimonas*, *Odoribacter*, *Parabacteroides*, *Oxalobacter*, *Phascolarctobacterium*, *Bilophila*, and *Ruminococcaceae UBA1819*) and four genera were under-represented (*Defluviitaleaceae UCG*-*011*, *Escherichia*-*Shigella*, *Faecalibaculum*, and *Alistipes*) (adj.*p* < 0.05) in FT mice (Figure 4).

### 2.2. Microbiota Dynamics During Tumor Growth (T2–T3)

Next, we investigated whether transplantation of human healthy microbiota affected the gut microbiota of mice bearing PDXs. The volume of PDXs was 80–150 mm^3^ at 4–8 weeks after implantation (early growth; T2), and 1000–2000 mm^3^ after 3–4 weeks (advanced growth; T3).

**Figure 5:**
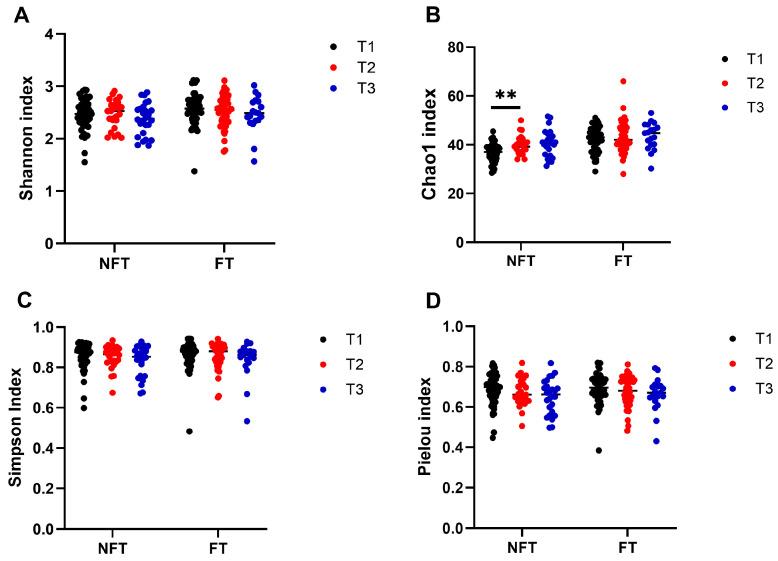
Comparison of α-diversity indices: Shannon (**A**), Chao1 (**B**), Simpson (**C**) and Pielou (**D**), in NFT and FT mice between T1 (*n* = 51/46) and T2 (*n* = 26/46) and between T2 and T3 (*n* = 26/19). The results were compiled from four separate experiments. Statistical significance: ** adjusted *p* < 0.01.

The Shannon, Simpson and Pielou indexes did not indicate changes in the response to tumor growth between T1 and T2 (Figure 5A,C,D), whereas the Chao1 index (Figure 5B) increased significantly only in the NFT group. There were no changes in either index between T2 and T3 in FT and NFT mice (Figure 5A–D).

**Figure 6:**
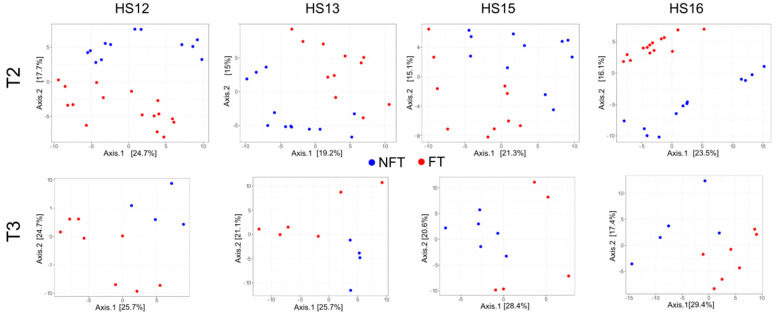
Principal coordinate analysis of fecal samples collected at T2 (upper panels) and T3 (lower panels). Each dot represents a single sample; NFT and FT mice are shown in blue and red, respectively.

As before, PCoA showed a clear separation of the β-diversity between FT and NFT mice in T2 (upper panel) and T3 (lower panel, Figure 6), with *p*-values = 0.0175 and 0.0165 as evaluated by PERMANOVA for T2 and T3, respectively.

Pairwise comparison between T1 (PDX grafting) and T2 (early phase of tumor growth) identified three genera (*Lachnospiraceae* NK4A136 group, FC = 0.49, adj.*p* = 2.82 × 10^−5^; *Lachnospiraceae* GCA-900066575, FC = 0.33, adj.*p* = 0.00097; [*Eubacterium*] *nodatum* group, FC = 1.69; adj.*p* = 0.0089) and six genera (*Marvinbryantia*, FC = 3.79, adj.*p* = 77 × 10^−6^; *Intestinimonas*, FC = 3.03, adj.*p* = 0.00011; *Roseburia*, FC = 2.81, adj.*p* = 0.0012; *Parasutterella*, FC = 0.41, *p* = 0.012; *Lachnospiraceae GCA-900066575*, FC = 0.55, adj.*p* = 0.021; *Bilophila*, FC = 1.67, adj.*p* = 0.041) with different abundance between the NFT and FT groups. Five genera (*Butyricimonas*, FC = 0.003, adj.*p* = 2.29 × 10*^−^*^86^; *Parabacteroides*, FC = 0.15; adj.*p* = 1.01 × 10^−11^; *Odoribacter*, FC = 0.056, adj.*p* = 1.67 × 10^−11^; *Oxalobacter*, FC = 0.11, adj.*p* = 1.02 × 10^−5^; *Defluviitaleaceae UCG-011*, FC = 4.87, adj.*p* = 5.66 × 10^−5^) that differed between FT and NFT mice at T2 were also included in the list of differential bacteria between the two groups at T1. At the final time point (T3), *Butyricimonas* (FC = 0.03, adj.*p* = 5.06236 × 10^−20^) and *Odoribacter* (FC = 0.03, adj.*p* = 2.72164 × 10^−7^) were significantly less abundant, whereas *Defluviitaleaceae UCG*-*011* (FC = 6.90, adj.*p* = 0.004) and *Escherichia*-*Shigella* (FC = 28.05, adj.*p* = 0.007) were more abundant in the gut microbiota of FT mice than in that of NFT mice.

### 2.3. FMT Modulates the Effects of FOLFOX Treatment

**Figure 7:**
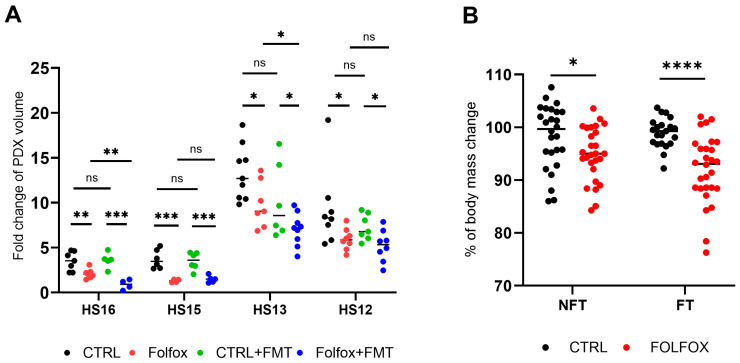
(**A**) Therapeutic effect of FOLFOX administered in 4–5 intraperitoneal injections at 5-day intervals in CRC PDX-bearing FT mice (transplanted with human healthy microbiota) and NFT mice. The effects were assessed by estimating the fold change in tumor volume at T3 compared with T2. The significance of differences between groups was tested separately for each experiment (n = 4–9 mice in each groups). (**B**) Percentage change in body weight from T2 to T3. The body weight was measured in each of four experiments and the average is shown (NFT, CTRL/FOLFOX, n = 26/27; FT, CTRL/FOLFOX, n = 21/28). Statistical significance: ns—*p* > 0.05; * *p* < 0.05; ** *p*  <  0.01; *** *p*  <  0.001; **** *p* < 0.0001.

FOLFOX treatment was started at T2, when implanted PDXs reached a volume of 80–150 mm^3^. The efficacy of treatment was determined by calculating the fold change in tumor volume between T2 and T3. FT had no effect on tumor growth in mice injected with saline in either of the four experiments performed using different PDXs. FOLFOX significantly inhibited tumor growth in all PDXs compared with the untreated controls, and FT potentiated the anti-tumorigenic activities of FOLFOX in two (HS13 and HS16) of the four experiments (Figure 7A). Body weight was significantly lower in FOLFOX-treated mice than in vehicle-treated mice, although the difference was not as marked in NFT as in FT mice (Figure 7B).

**Figure 8:**
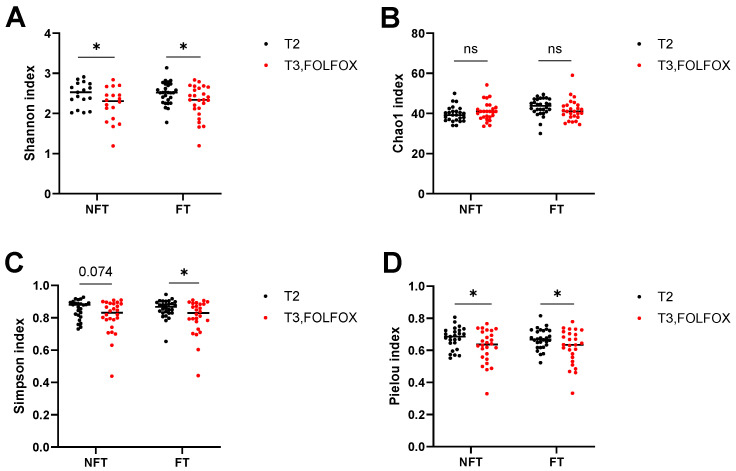
Comparison of α-diversity indices: Shannon (**A**), Chao1 (**B**), Simpson (**C**) and Pielou (**D**), in FOLFOX-treated NFT and FT mice between T2 and T3. The results were compiled from four separate experiments (*n* = 26). Statistical significance: ns—*p* > 0.05; * adjusted *p* < 0.05.

At the T3 time point, FOLFOX treatment decreased the Shannon, Simpson and Pielou indexes, and did not change the Chao1 index in both FT and NFT mice (Figure 8A–D) as compared to T2.

**Figure 9:**
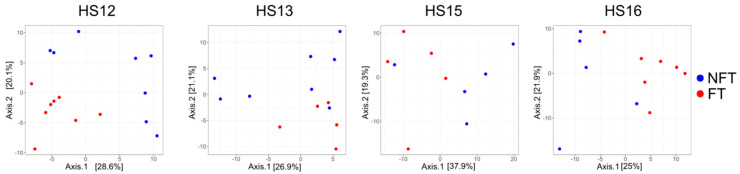
Principal coordinate analysis of fecal samples collected at the end of experiments (T3) from FOLFOX-treated mice. Each dot represents a single sample, and NFT and FT mice are shown in blue and red, respectively.

PCoA showed significant differences in β-diversity between FT and NFT mice treated with FOLFOX at T3 in HS12, HS13, HS15, and H16 experiments (Figure 9), with *p* value = 0.0001.

**Figure 10:**
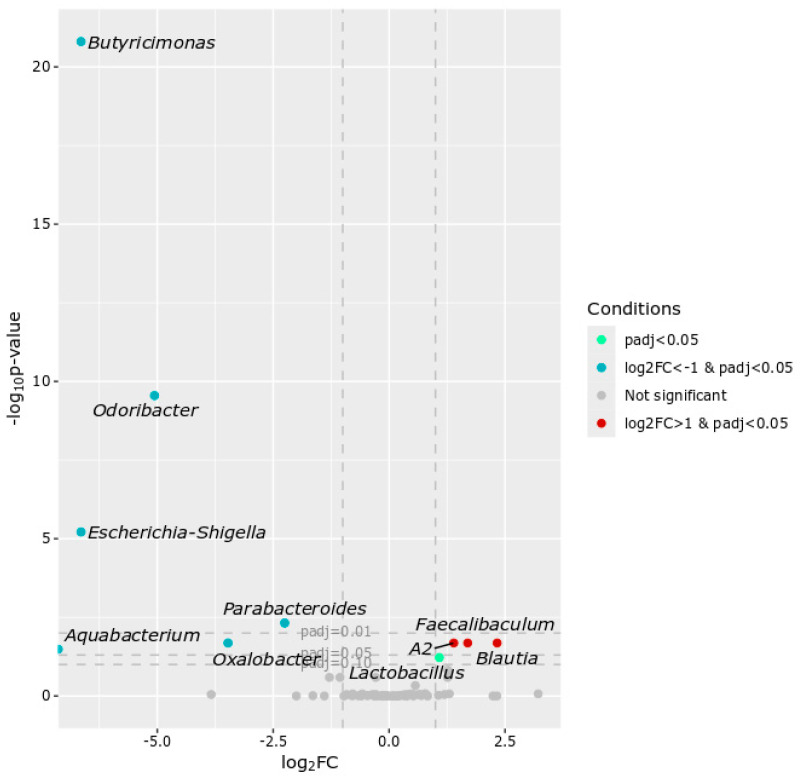
Volcano plot showing differential abundance of taxa. Each point represents a taxon, plotted by log2 fold change (log2FC) and statistical significance (−log10 *p*-value). The vertical grey dotted lines indicate the log2 fold change (log2FC) thresholds of −1 and 1.

Taxonomic analysis of the intestinal microbiota from FOLFOX-treated mice at the T3 point identified 10 genera that differed between FT and NFT mice (Figure 10). Of these, *Butyricimonas*, *Odoribacter*, *Escherichia*-*Shigella*, *Parabacteroides*, *Oxalobacter* and *Aquabacterium* were over-represented, whereas *Faecalibaculum*, *Lachnospiraceae A2*, *Blautia*, and *Lactobacillus* were under-represented in FT mice (Figure 10).

### 2.4. Minimal Effects of FMT on Stool Metabolites

**Figure 11:**
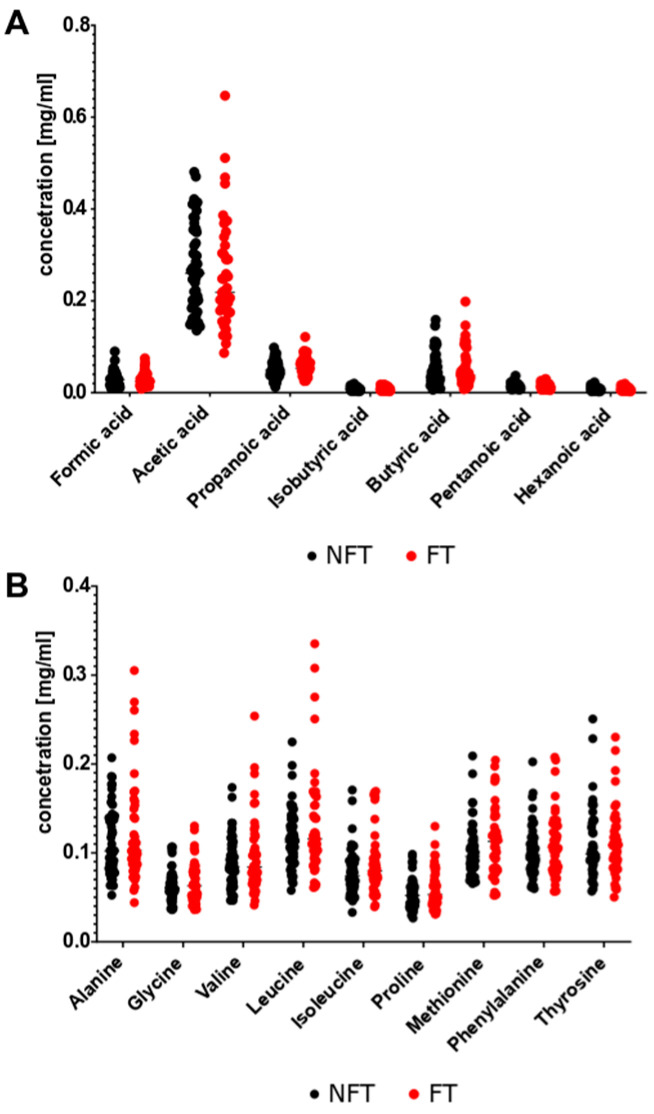
Relative abundance of SCFAs (**A**) and amino acids (**B**) in NFT (*n* = 50) and FT (*n* = 47) mice at 2 months after transplantation (T1). The results were compiled from four separate experiments.

**Figure 12:**
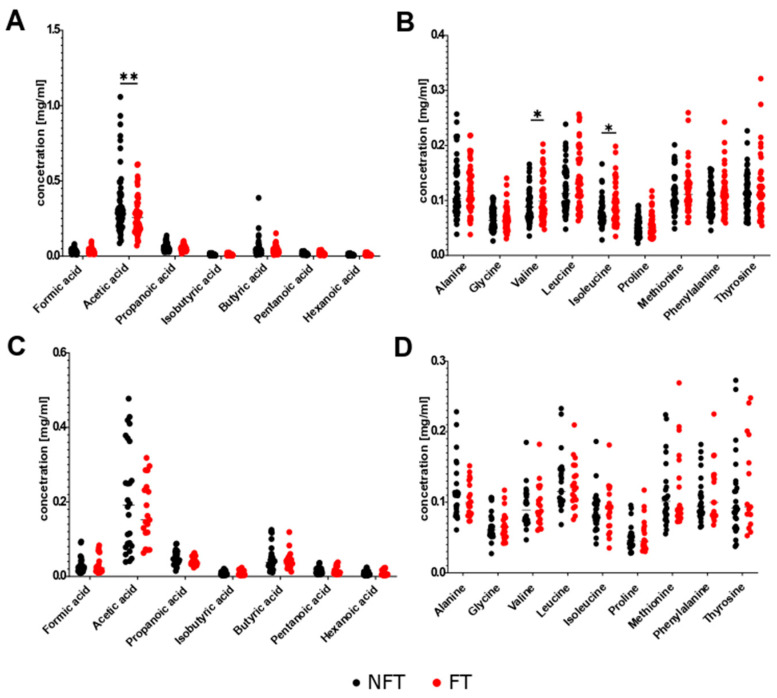
Relative abundance of SCFAs (**A**,**C**) and AAs (**B**,**D**) in NFT (*n* = 50) and FT (*n* = 46) mice at T2 (**A**,**B**) and at T3 (*n* = 26/19) (**C**,**D**). The results were compiled from four separate experiments. Statistical significance: * *p* < 0.05; ** *p*  <  0.01.

**Figure 13:**
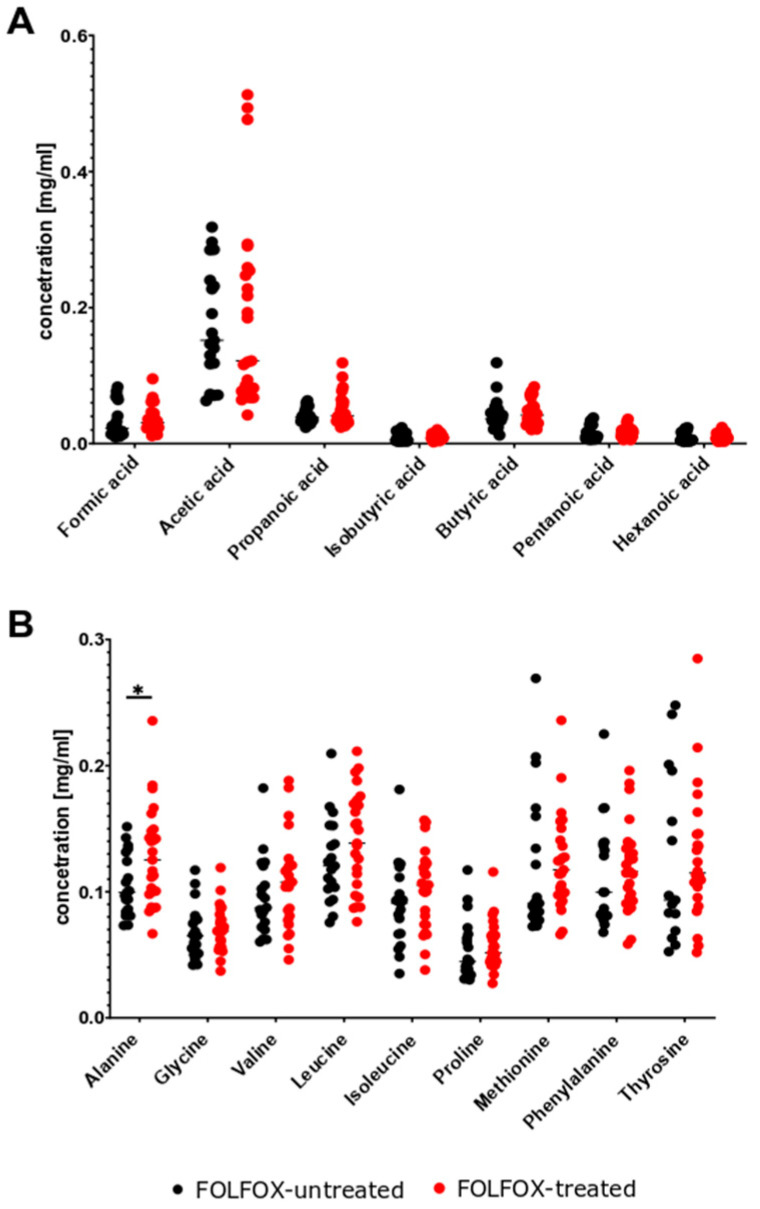
Relative abundance of SCFAs (**A**) and AAs (**B**) in FOLFOX-treated (*n* = 25) and untreated mice (*n* = 19) transplanted with human fecal microbiota. The results were compiled from four separate experiments. Statistical significance: * *p* < 0.05.

Seven SCFAs and nine AA were analyzed in fecal samples collected at different time points throughout the experiment. No differences in the levels of these metabolites were detected between stool samples collected from FT and NFT mice at T1 (Figure 11). Pairwise comparison at T2 (early tumor growth phase) showed a significant decrease in acetic acid levels and increase in valine and isoleucine levels in FT mice (Figure 12A,B). However, FT had no effect on the levels of the metabolites analyzed at T3 after considerable tumor growth (Figure 12C,D). There were no changes in metabolite levels in response to FOLFOX treatment in stool samples from FT and NFT mice, except one case that showed an increase in alanine levels in FOLFOX-treated FT mice compared with untreated FT mice (Figure 13A,B).

## 3. Discussion

The intestinal microbiota can modulate metabolic, inflammatory, and immune responses in cancer patients. Gut dysbiosis plays an important role in cancer development and prevention, and the association of gut microbiota dysbiosis with CRC has been studied extensively [18]. Among the factors that modulate gut microbial communities, diet, probiotics, antibiotics, and FMT have received the most attention [19]. In this study, NSG mice grafted with four different CRC PDXs were used to investigate the potential impact of FMT on tumor growth and the response to FOLFOX chemotherapy. Fecal microbial communities were assessed using 16S rRNA metagenomic sequencing, and targeted fecal metabolomic profiles (SCFAs and AAs) were tested using GC/MS approaches.

FMT in cancer treatment involves the transfer of healthy gut microbiota to restore a diverse and beneficial gut ecosystem that may be crucial to enhance the effectiveness of therapy and potentially reduce treatment-related side effects. Therefore, the selection of donors for FMT focuses on their good general health; healthy BMI; healthy diet and lifestyle; absence of chronic GI disorders, autoimmune diseases or cancer; absence of recent treatment with antibiotics; and probiotics.

To maximize the likelihood of successful engraftment and minimize the variability associated with individual donor characteristics, we used pooled stool samples from eight healthy donors instead of material from a single donor. Mice underwent intragastric administration of human pooled stool at weekly intervals for a total of 12 treatments, preceded by intestinal cleansing with PEG solution at monthly intervals. This recommended procedure may stably colonize the mouse intestines with the human microbiome [13]. NFT (control) mice received saline vehicle by intragastric administration. At the beginning of the experiment, mice aged 7–9 weeks were considered to be in the maturing stage, and two months later they were already mature mice. Consistent with the changes in intestinal microbiota communities that occur in the early life phases in humans and animals [20], we found 16 differentially abundant genera between maturing and mature mice that were common to the NFT and FT groups. Of these, members of the *Lachnospiraceae* family have anti-inflammatory and antitumorigenic properties [21], and *Alistipes* (consisting of 13 species) exhibit protective effects against diseases such as colitis [22].

The reduced weight gain in FT mice likely resulted from the FMT procedure, which involved intensive intestinal cleansing with PEG and repeated gavage. Importantly, FMT alone did not influence tumor growth (Section 2.3), and in our previous study using the same PDX platform [23], FOLFOX did not induce significant toxicity or weight loss, which could confirm that the observed changes were not related to FOLFOX therapy. All animals were monitored with humane endpoints and no differences in survival were observed. Future work should include pharmacokinetic profiling to confirm these findings.

In this study, FMT modified the gut bacterial community structure in FT groups, including an increase in α-diversity indexes and significant differences in β-diversity. Taxonomic analyses identified 11 genera, of which seven were over-represented (*Butyricimonas*, *Odoribacter*, *Parabacteroides*, *Oxalobacter*, *Phascolarctobacterium, Bilophila, UBA1819*) and four were under-represented (*Defluviitaleaceae UCG-011*, *Escherichia-Shigella*, *Faecalibaculum*, *Alistipes*) in FT mice compared with NFT mice.

The pro- or anti-tumor effects of specific bacterial strains or gut microbiota-related metabolites have been highlighted in many human and animal studies [11]. The presence of a grafted tumor significantly increased the microbiota diversity and slightly changed the microbiota composition, including altering the abundance of *Lachnospiraceae*, *Clostridiaceae*, *Ruminococcaceae*, and *Streptococcaceae* [24]. ApcMin/+ mice transplanted with feces from CRC patients developed high-grade dysplasia and an increase in the number of intestinal tumors, and *Lactobacillus*, *Bifidobacterium*, non-enterotoxigenic *Bacteroides fragilis* (NTBF), and *Faecalibaculum rodentium* were considered to have anticancer activities [12]. In MB49 tumor-bearing mice, delivery of *Parabacteroides distasonis* enhanced the effect of anti-PD-1 immunotherapy [11]. *Escherichia-Shigella* was strongly correlated with a normal post-chemotherapy white blood cell count compared with the post-chemotherapy hypoleukocyte patient group [25]. FMT with *Eubacterium rectale*, *E. eligens*, *E. ventriosum*, and *Collinsella aerofaciens* suppresses tumor growth in a humanized breast cancer model [26]. Pre-treatment with *Lactobacillus* before the establishment of tumors in colon cancer models suppressed tumor formation, and continuous oral administration of living *Lactobacillus* significantly suppressed tumor growth [27]. A potential role for *Akkermansia*, *Anaeroplasma*, and *Alistipes* in regulating colonic inflammation in inflammation-driven CRCs was suggested [28]. The abundance of *Alistipes* was higher in mice receiving naringenin treatment than in the control and untreated groups [10]. Enrichment of *Alistipes* sp. and *Odoribacter splanchnicus* was also noted in patients with ulcerative colitis in clinical remission after FMT, whereas *Escherichia coli* and *Klebsiella* were enriched in those who did not achieve remission [29]. The genus *Escherichia-Shigella* is over-represented in patients who do not respond to immune checkpoint inhibitor therapy [30]. *Lactobacillus* decreases tumor load, the level of inflammation, and DNA damage [13].

In this study, transplantation of healthy human microbiota did not modify the growth of PDXs and did not change the bacterial α-diversity in the early or advanced phase of tumor growth with one exception: the Chao1 index increased during early tumor growth in NFT mice, and FMT potentiated this increase. Inter-group differences in microbial communities analyzed by β-diversity showed separate clusters in FT and NFT mice with both early and advanced tumors. The relative abundance of the genera *Butyricimonas*, *Parabacteroides*, *Odoribacter*, *Oxalobacter*, and *Defluviitaleaceae UCG-011* distinguished FT from NFT mice at the early phase of tumor growth. However, there were no differences in bacterial genera in the intestinal microbiota between early and advanced tumor growth stages in both NFT and FT mice groups. These results suggest that the changes in bacterial communities were caused by the FMT itself and were not related to the presence of tumors.

Pooling donors is commonly recommended in preclinical FMT research as it increases microbial diversity, reduces donor-specific bias, and provides a more representative ‘healthy’ microbiota profile [31,32]. However, a key limitation of pooling is that donor-specific persistence cannot be tracked. In this study, FMT engraftment was assessed based on α- and β-diversity and taxonomic differences between FT and NFT mice. Future studies using strain-level metagenomics or defined microbial consortia are needed to identify specific taxa or microbial functions that modulate chemotherapy effectiveness.

The modulatory effects of FMT have been studied in various pathologies linked to gut bacterial dysbiosis, such as inflammatory bowel diseases, obesity, and toxicity induced by chemotherapy, immunotherapy, and radiotherapy [13]. In this study, FOLFOX significantly decreased mouse body weight, and the effect was less pronounced in NFT than in FT mice and prevented the growth of grafted PDXs; FMT potentiated this effect in two of the four PDXs. In FOLFOX-treated mice, FMT did not change the Shannon index, increased the Chao1 index, altered bacterial β-diversity, increased the abundance of *Butyricimonas*, *Odoribacter*, *Escherichia-Shigella*, *Parabacteroides*, *Oxalobacter*, and *Aquabacterium*, and decreased that of *Faecalibaculum*, *Lachnospiraceae A2*, *Blautia*, and *Lactobacillus*.

Previous reports showed the following findings: Oral–fecal microbiota transplantation improved dysbiosis related to FOLFOX chemotherapy in mice [4]. FMT restored 5-FU-induced gut dysbiosis, richness, and diversity in a healthy mouse model [33]. In tumor-bearing mice, β-diversity indexes did not significantly differ between saline, FMT, FOLFOX, and FOLFOX + FMT groups, whereas α-diversity showed compositional changes in the fecal microbiota used for FMT in the FOLFOX + FMT group [13]. In mice subcutaneously implanted with syngeneic CT26 colorectal adenocarcinoma cells, preventive administration of *Lactobacillus casei* Variety *rhamnosus* dose-dependently reduced the severity of FOLFOX-related diarrhea and intestinal mucositis without affecting its antitumor effect [34]. It is important to note that the PDX tumors in this study were implanted subcutaneously, not orthotopically in the colon. Therefore, intestinal tissue was not involved in tumor growth or treatment assessment. In colorectal tumor-bearing mice, the combination of anti-PD-1 therapy and FMT resulted in a higher survival rate and better tumor control than anti-PD-1 therapy or FMT alone, with an increase in *Bacteroides thetaiotaomicron* and *B. fragilis* and a decrease in *B. ovatus* after FMT [35]. FMT from healthy mice significantly attenuated liver metastasis in microbiota-depleted nude mice [36]. Pretreatment with an oral antibiotics cocktail integrated with bacterial gavage (*Staphylococcus*, *Jeotgalicoccus*, *Sphingomonas*, and *Prevotella*) alleviated the decrease in FOLFOX-related body weight in xenografted animals, whereas the therapeutic effect of FOLFOX was suppressed only in response *Prevotella* colonization [37]. FMT reversed intestinal microbial dysbiosis in CRC mice [13].

Although FMT has been evaluated in different animal disease models and clinical trials, the mechanisms underlying the beneficial effects of the transfer of fecal material remain unknown. Some mechanisms have been proposed, such as restoration of the disturbed intestinal microenvironment and direct interaction of donor gut microorganisms and their active products with the host’s gut mucosal barrier and immune system.

In this study, four different CRC PDX models exhibited different growth dynamics as well as different genetic and transcriptomic profiles which assigned HS12, HS13, H15 and HS16 models to the PDX classifier 3, 1, 4 and 2, respectively [38]. While the experiments spanned a two-year period, and followed identical protocols (diet, housing, FMT procedure), the control groups in HS12 and HS13 showed greater variability than those in HS15 and HS16, likely reflecting natural differences in endogenous microbiota. This variability did not alter the main conclusions, as trends in α- and β-diversity and metabolite profiles remained consistent across experiments (Figure 3 and Figure 6).

The effect of FMT on the FOLFOX response was model-specific, being observed in two out of four PDX models. Although the HS12 and HS16 experiments showed more pronounced separation in the β-diversity analysis between the FT and NFT groups, the enhancement of the efficacy of FOLFOX was observed in HS13 and HS16 models. We conclude, therefore, that the magnitude of β-diversity shifts does not necessarily predict functional outcomes such as chemotherapy response which, in turn, may depend on specific host-microbiota-tumor interactions rather than on the general extent of microbiota restructuring.

PDX models, while powerful for simulating human tumors, have several specific limitations: (1). The use of immunodeficient mice prevents the study of human immune responses; (2). Human stromal cells are replaced by mouse counterparts, altering crucial tumor-stroma interactions; (3). Genetic diversity within a tumor can be lost during serial passing, failing to capture the full complexity of the original cancer; (4). The success rates of engraftment vary by cancer type, and only the most robust tumor cells survive and grow in the mouse, potentially selecting more aggressive clones [38,39]. The translational relevance of our findings is mostly limited using immunodeficient mice, as NSG mice lack functional T, B and NK cells, which prevents immune-mediated mechanisms that are critical for response to chemotherapy and microbiota–host interactions in patients. Consequently, the potential immunomodulatory effects of FMT observed in clinical settings could not be captured in this model. Furthermore, although FMT induced robust microbiota remodeling, recipient mice retained murine-specific gut physiology and immune signaling, which differs from the human intestinal environment. Another important limitation is the difference in tumor biology: human CRC develops over decades, mainly through a multistep adenoma-carcinoma sequence, whereas PDX tumors in mice exhibit rapid growth within weeks. This accelerated timeline may not recapitulate the chronic interplay between the microbiota, immune system, and tumor evolution observed in patients. Therefore, our results should be interpreted as proof-of-concept rather than directly extrapolated to clinical settings. Future studies should incorporate immune-competent or humanized immune models to better mimic the patient’s physiology and validate the mechanistic links suggested by our data.

Body weight loss after FOLFOX was consistent with previous reports of chemotherapy-induced stress in xenograft models, but our previous work using the same PDX platform showed that FOLFOX does not induce significant systemic toxicity in NSG mice [23]. Except for daily monitoring according to humane endpoints, no specific endpoints for chemotherapy toxicity were evaluated, such as multiorgan toxicity, RBC counts or chemotherapy-related fatigue [40].

Differences in the abundance of bacterial groups can change the metabolic function of the gut microbiota [41]. Of the metabolites generated by the intestinal microbiota, SCFAs are the most abundant. SCFAs are produced by the fermentation of indigestible carbohydrates by commensals such as *Faecalibacterium prausnitzii*, *Roseburia intestinalis*, and *Anaerostipes butyraticus*, and maintain intestinal homeostasis in the normal colon [42]. Acetate, which is mostly produced by *Bifidobacteria* spp., maintains gut–epithelial barrier function and regulates intestinal inflammation, whereas butyrate acts as an important energy source for colonocytes and maintains gut barrier homeostasis [43,44]. Bacterial metabolic processes in distal parts of the colon may be related to the availability of AAs [45]. In this study, a targeted stool metabolomics panel remained largely unchanged in all experiments (Section 2.4). However, we did not include formal correlation analyses between metabolite levels and chemotherapy outcomes due to transient changes in T2, lack of persistence in T3, and limited statistical power. These findings suggest that compositional changes alone are insufficient to drive chemotherapy potentiation. However, it should be noted that the metabolomics analysis was limited to seven SCFAs and nine AAs (Section 2.4; Figure 11, Figure 12 and Figure 13), while other metabolites were not analyzed and therefore more studies are needed to identify metabolites that might mediate effects of chemotherapy.

Another consideration is the expected relationship between microbiota compositional changes and microbiota-derived metabolites, and the discrepancy between our findings and previous studies linking SCFAs to chemotherapy efficacy. Our targeted metabolomics revealed minimal or transient SCFA changes despite robust microbiota alterations, which likely reflects differences in the experimental context. Most SCFA-related effects have been reported in immunocompetent models, where SCFAs modulate mucosal immunity and inflammation [46,47]. In contrast, NSG mice lack adaptive immunity, and PDX tumors grow in a profoundly immunodeficient environment, which likely attenuates SCFA-mediated mechanisms. FMT-associated immune activation described in such models cannot be fully recapitulated in NSG mice due to their lack of functional T, B, and NK cells. These observations suggest that the FMT-mediated modulation of FOLFOX efficacy in our study is not driven by SCFA levels but may involve other microbiota–host interactions, such as drug metabolism or microbial signaling pathways. Thus, although FMT induced marked compositional changes in the gut microbiota, the lack of a functional immune system in NSG mice likely uncouples microbiota alterations from downstream immune-dependent metabolic effects. Therefore, future studies should integrate untargeted metabolomics and immune-competent models to clarify these mechanisms.

## 4. Materials and Methods

### 4.1. Experimental Design Overview

Between 2022 and 2023, four independent experiments (HS12, HS13, HS15, and HS16) were conducted using four different colorectal cancer (CRC) patient-derived xenograft (PDX) models grafted onto NSG mice. A total of 98 animals completed the full experimental protocol, including fecal microbiota transplantation and FOLFOX chemotherapy: 51 mice were transplanted with human fecal microbiota, and 47 served as non-transplanted controls. Mice were aged 7–9 weeks with a mean body weight 24.8 ± 3.6 g at the time of randomization into study or control groups and were maintained on a standard (normal) diet (4.4 g fat, 16.5 g protein, and 70.6 g carbohydrate in 100 g dry weight feed, with a metabolic energy content of 16.38036 MJ (Research Diets, Inc., New Brunswick, NJ, USA). Body weight was monitored weekly with a precision of 0.1 g throughout the experiment.

The group sizes used in this study were estimated based on prior experimental experience and verified using statistical power analysis. A Student’s *t*-test was used to assess effect size and to confirm that the chosen group sizes were sufficient to detect statistically significant differences with a test power of 0.8 and a significance level of α = 0.05. The selected group sizes were considered appropriate for the experimental design.

### 4.2. Ethical Approval and Animal Welfare Procedures

All animal procedures were conducted in accordance with the EU Directive 2010/63/EU and were approved by the 2nd Local Ethics Committee for Animal Experimentation in Warsaw (approval number WAW2/117/ 2018, issued on 25 July 2018). Animals were housed under specific pathogen-free conditions with controlled temperature, humidity, and a 12 h light/dark cycle, with ad libitum access to food and water. Each cage contained 3–5 mice.

Animals were monitored daily for signs of distress, including changes in behavior or appearance. Humane endpoints were predefined in the approved protocol and included body weight loss, piloerection, vocalization, or abnormal posture (e.g., hunched or arched back). Mice meeting these criteria were euthanized following veterinarian assessment. Euthanasia was performed by cervical dislocation under deep inhalation anesthesia (5% isoflurane; Baxter, Deerfield, IL, USA) to ensure full unconsciousness prior to the procedure. Oral gavage procedures and tumor implantation were performed under short-term or general inhalation anesthesia (2–5% isoflurane) to minimize pain and distress.

### 4.3. Fecal Microbiota Transplantation (FMT) Protocol

The NSG mice were purchased from the Jackson Laboratory in Bar Harbor, ME, USA, and kept in suitable environment conditions (temperature, humidity, and 12 h light cycles) and were free to access water and food. Each cage contained 3–5 mice. Two weeks before transplantation, mice between 5 and 6 weeks of age were transferred from breeding to experimental research area in animal breeding facilities. On the day of FMT, mice were transferred to clean cages to avoid coprophagia, and for 1 h they were denied access to food. Afterwards, mice were randomly assigned to research and control groups. The mice of the study group received a water solution of 200 µL polyethylene glycol (PEG) (Macrogol 4000; Sigma-Aldrich, St. Louis, MO, USA) at a concentration of 425 g/L by gavage three times over a 20 min interval. The procedure was performed with 2% isoflurane in a short anesthesia. A period of 4 h after PEG’s last intragastric administration, 200 µL of human feces suspension was administered intragastrically to the experimental mice, while the control group received 200 µL of saline. The intragastric administration of the fecal suspension was repeated three times at weekly intervals (first round of transplantation). Overall, the transplantation procedure was repeated three times.

Animals were monitored daily for health status. Body weight was measured weekly during stool collection and compared to baseline values. Animals showing signs of distress were euthanized as per the humane endpoints described above.

### 4.4. Preparation of the Human Fecal Suspension

Eight healthy, lean individuals (four women and four men aged 18–29 years, with a body mass index (BMI) below 25 kg/m^2^, no chronic diseases and no recent antibiotic use) were recruited as stool donors in July 2018, in accordance with the decision of the Local Bioethics Committee of the Maria Skłodowska-Curie National Research Institute of Oncology (Decision 54/2017). Fecal samples were self-collected using a stool specimen collection kit, as described previously [3]. Following collection, the fecal suspension was immediately prepared and stored at −80 °C until use. A gram of stool samples from each donor was suspended in 40 mL of PBS (1 g of stool/5 mL of PBS; Thermo Fisher Scientific, Waltham, MA, USA). The suspension was homogenized and centrifuged (1500 rpm), and the supernatants were filtered through 100 µm Millipore filters (Merck Millipore, Burlington, MA, USA). Glycerol was added to the filtrate to a final concentration of 10%, and aliquots were stored at −80 °C. Prior to administering to mice, the suspension was diluted four times with sterile water.

### 4.5. Tumor Engraftment

Four CRC PDX models, developed at Maria Sklodowska-Curie National Institute of Oncology in NU/J mice (generation P3–P7) and cryopreserved in a medium with 50% DMEM, 40% FBS and 10% DMSO, were used [38]. After thawing, tumor pieces were implanted subcutaneously on the flank of two to four NSG mice between 6 and 10 weeks old under general inhalation anesthesia. Tumor fragments were implanted subcutaneously on the flank of NSG mice, which is standard for patient-derived xenograft (PDX) models and enables accurate monitoring of tumor growth using external calipers. When the tumor volume reaches 1000–1500 mm^3^, the tumor tissue is cut and placed on the left side of the mouse to begin the third FT round. When tumors exceed 80–150 mm^3^, animals are randomly assigned to FOLFOX-treated and non-treated (control) groups. Of 128 mice included in these studies, 30 animals were excluded from the analysis due no tumor growth or excessive tumor growth that exceeded humane endpoint criteria.

### 4.6. Administration of FOLFOX Chemotherapy

The FOLFOX regimen included intraperitoneal injections of oxaliplatin (6 mg/kg) (Oxaliplatin Kabi, Fresenius Kabi, Poland), 5-FU (50 mg/kg) (5-Fluorouracil, Ebewe Pharma Ges.m.b.H Nfg. KG, Unterach am Attersee, Austria) and folinic acid (90 mg/kg) (Levofolic, medac Gesellschaft für clinicische Spezialpräparate mbH Theaterstr., Wedel, Germany) administered every 5 days. Control animals received an equivalent volume of 0.9% saline. Tumor volume was measured weekly with calipers and calculated using the formula: (length × width × width)/2.

Anesthesia or analgesia was not used during IP injections or tumor measurements, as these procedures were brief and the use of such agents was judged to cause greater distress than the procedures themselves. This decision was approved by the local ethics committee.

Animals were euthanized after the fourth or fifth FOLFOX administration if early signs of distress appeared, following the humane endpoint protocol. Euthanasia was performed by cervical dislocation under deep inhalation anesthesia (5% isoflurane), and necropsy was conducted for tissue and organ collection.

### 4.7. 16S-rRNA Sequencing and Metabolomics Procedures

Collected fecal samples were stored at −80 °C until used. Bacterial DNA was isolated from mouse fecal samples using a QIAamp Fast DNA Stool Mini Kit (Qiagen, Hilden, Germany) according to the manufacturer’s protocol, as described previously [48]. The quality and quantity of the extracted DNA were assessed by measuring the optical density using a NanoDrop 2000/2000c spectrophotometer (Thermo Fisher Scientific, Carlsbad, CA, USA) and a fluorometric-based method using a Qubit dsDNA HS Assay Kit (Thermo Fisher Scientific), respectively. Library preparation of the variable V3 and V4 regions of the bacterial 16S rRNA gene was performed according to the 16S Metagenomic Sequencing Library Preparation protocol on an Illumina platform (Illumina, Inc., San Diego, CA, USA). Sequences were obtained on an Illumina MiSeq system in a 2 × 300 bp paired-end run.

SCFAs and AAs were extracted and derivatized as described previously [49,50]. Gas chromatographic analysis of fecal extracts was performed on an Agilent 7000D Triple Quadrupole mass spectrometer coupled with a 7890-gas chromatography (GC) system with a G4513A autosampler (Agilent Technologies, Santa Clara, CA, USA). A VF-5ms column (30 m, 0.25 mm, 0.50 µm) was used for analysis. Mass spectrometry (MS) data were collected in full scan mode for m/z 15–650 at a frequency of 4.9 scans per second. MassHunter software (version B.07.00, Agilent Technologies, Santa Clara, CA, USA) was used for analysis.

**Figure 14:**
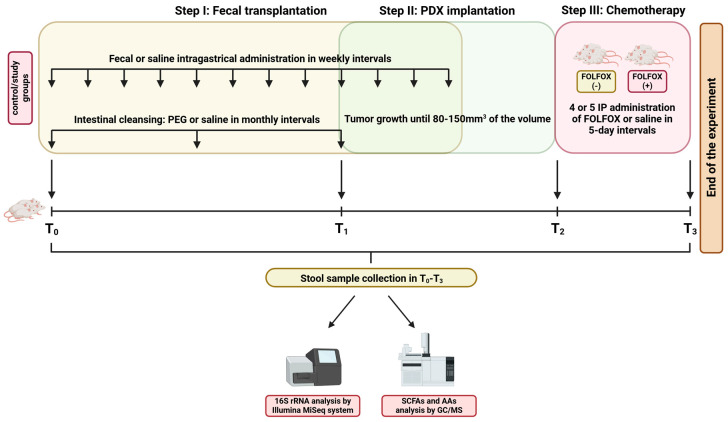
Graphical summary of the study design. On the first day of the experiment (time 0; T0), mice underwent intestinal cleansing with a PEG solution and intragastric administration of the water extract of pooled stools from healthy humans by oral gavage. Control mice received sterile saline. Intestinal cleansing was repeated monthly, and fecal extract administration was performed at weekly intervals. After 2 months, at the beginning of the third cycle of fecal transplantation, pieces of freshly resected PDXs were subcutaneously implanted into fecal transplanted (FT) and not fecal transplanted (NFT) mice. When the tumor volume reached 80–150 mm^3^, animals were assigned randomly to FOLFOX-treated and -untreated groups (*n* = 4–8 mice per group). Stool samples were collected and frozen at −80 °C at T0, before PDX engraftment (T1), before FOLFOX treatment was started (T2), and on the day the mice were euthanized (T3). Four separate experiments using different CRC xenografts were performed.

The experimental procedure used in this study is illustrated in Figure 14.

### 4.8. Statistical Methods

DADA2 24 pipeline version 1.26 [51] was used for read error correction, amplicon sequence variant identification, and chimeric read identification and removal. Taxonomy assignment was performed with Mothur version 1.43 [52] using SILVA (version 1.138 26) [53]. Diversity indices (Shannon, Chao1, Simpson and Pielou’s evenness index) were computed with the estimate richness function from the phyloseq R package version 1.46.0 (Bioconductor, Stanford University, Stanford, CA, USA) [54]. The rarefaction depth used in the diversity analyses was 5000. Significant differences between groups were identified with the Mann–Whitney U test or mixed-effects linear models (as implemented in package lmerTest version 3.1 [55]). β-diversity differences were tested using PERMANOVA implemented in the adonis2 function from the vagan package (version 2.6-6.1) [56]. A separate “~FMT + batch” model was used for each timepoint and control/FOLFOX treatment. The Bray–Curtis distance was used and the number of permutations was 9999. Differentially abundant amplicon sequence variants were identified with DEseq2 version 1.42.0 (Bioconductor, Heidelberg, Germany) (on normalized data), with *p*-values corrected using the Benjamini–Hochberg [57] procedure to minimize the false discovery rate. Analyses were performed using R, version 4.3.2.

Body weight, tumor volumes, and relative abundances of AAs and SCFAs between groups were compared using multiple two-sample t tests with the “two-stage” Benjamini, Krieger, & Yekutieli [58] procedure to control the false discovery rate (FDR) of a family of hypothesis tests, employed by GraphPad, v8.2.1 (GraphPad Software, San Diego, CA, USA).

## 5. Conclusions

Prolonged FMT in NSG mice substantially remodeled gut microbiota without altering fecal SCFA or AA profiles. While FMT did not influence baseline tumor growth, it enhanced the efficacy of FOLFOX in select CRC PDXs. Further studies should focus on optimizing FMT protocols and elucidating microbiota-derived mechanisms that modulate chemotherapy responses.

## Data Availability

The datasets presented in this study can be found in online repositories. The names of the repositories and accession numbers can be found below: https://www.ncbi.nlm.nih.gov/bioproject/1268737 and 1199764 (accessed on 28 May 2025).

## Supplementary Materials

The following supporting information can be downloaded at: https://www.mdpi.com/article/10.3390/ijms27031438/s1.

## Abbreviations

The following abbreviations are used in this manuscript:

CRC	Colorectal cancer
FMT	Fecal microbiota transplantation
PDX	Patient-derived xenograft
SCFAs	Short-chain fatty acids
AAs	Amino acids
FT	Fecal transplanted
NFT	No fecal transplanted
NSG mice	NOD scid gamma mouse
T0	experiment start time point
T1	time point after two months of fecal microbiota transplantation
T2	early growth time point
T3	advanced growth time point
PCoA	principal co-ordinate analysis
